# Performance Evaluation of Three Diagnostic Methods for Soil-Transmitted Helminth Infections among Schoolchildren in Amhara Region, Northwest Ethiopia

**DOI:** 10.1155/2023/9697165

**Published:** 2023-06-26

**Authors:** Shegaw Belay, Getaneh Alemu, Tadesse Hailu

**Affiliations:** ^1^Amhara National Regional State Health Bureau, Bahir Dar Health Science College, Bahir Dar, Ethiopia; ^2^Departments of Medical Laboratory Science, College of Medicine and Health Sciences, Bahir Dar University, Bahir Dar, Ethiopia

## Abstract

**Background:**

Soil-transmitted helminths are parasitic nematodes found in the intestine. They are more prevalent in the tropics and subtropics, including Ethiopia. However, low-sensitive direct wet mount microscopy fails to detect soil-transmitted helminths among infected cases. Therefore, more sensitive and cost-effective diagnostic methods are urgently needed to minimize soil-transmitted helminthiasis morbidity.

**Objective:**

This study aimed to compare and evaluate the performance of diagnostic methods for soil-transmitted helminths against the “gold” standard.

**Methods:**

An institution-based cross-sectional study was conducted among 421 schoolchildren from May to July, 2022 in the Amhara Region. Study participants were selected using a systematic random sampling technique. Stool samples were processed via Kato–Katz, McMaster, and spontaneous tube sedimentation techniques. Data were entered into epi-data version 3.1 and analyzed using SPSS version 25. The sensitivity, specificity, positive predictive value, and negative predictive value were calculated against the combined result as a “gold” standard. The strength of agreement between the diagnostic methods was determined by the Kappa value.

**Results:**

The overall prevalence of soil-transmitted helminths was 32.8% (95% CI: 28.2–37.8%) using a combination of methods. The detection rates of Kato–Katz, McMaster, and spontaneous tube sedimentation were 28.5% (95% CI: 24.2–33.2%), 30% (95% CI: 25.6–34.8%), and 30.5% (95% CI: 26.1–35.3%), respectively. Sensitivity and negative predictive values were 87.1% (95% CI: 80.2–92.3%) and 95.1% (95% CI: 92.6–96.8%) for Kato–Katz; 91.7% (95% CI: 85.6–95.6%) and 96.5% (95% CI: 94.1–98.0%) for McMaster; and 93.2% (95% CI: 87.5–96.8%) and 97.1% (95% CI: 94.7–98.4%) for spontaneous tube sedimentation. Kappa values of Kato–Katz, McMaster, and spontaneous tube sedimentation for the diagnosis of soil-transmitted helminths were 0.901, 0.937, and 0.948, respectively.

**Conclusion:**

Kato–Katz, McMaster, and spontaneous tube sedimentation techniques had comparable sensitivity with almost perfect agreement for the detection of soil-transmitted helminths. Therefore, the spontaneous tube sedimentation technique can be used as an alternative diagnostic method for soil-transmitted helminth infections in endemic countries.

## 1. Introduction

Soil-transmitted helminths (STHs) are nematodes that need soil for their infective eggs or larvae to develop. These include *Ascaris lumbricoides*, *Ancylostoma doudenale*, *Necator americanus*, and *Trichiuris trichura*. Soil-transmitted helminthiasis is among the most common infections worldwide and mainly affects the poorest and most deprived communities where people have poor nutrition, inadequate sanitation, live in an overcrowded environment, walk barefoot, and lack clean drinking water [1, 2].

According to a recent report, more than 4.5 billion individuals are at risk of infection, with more than 1.5 billion people being infected by STHs in the world [3]. Tropical and subtropical areas have the highest rates of infections, with sub-Saharan Africa, China, and East Asia having the highest infection rates. Over 835 million children (267 million preschool-aged children (PSAC) and 568 million school-aged children (SAC)) live in areas where these parasites are widespread and require preventive chemotherapy [4].

In Ethiopia, out of an estimated 112.1 million national population, more than 96.7 million people, which are comprised of 56.1 million adults, 27.7 million SAC, and 12.9 million PSAC are expected to live in STHs endemic areas. According to the current protocol, 68.1 million people (39 million adults, 19.8 million SAC, and 9.3 million PSAC) require treatment [5]. Moreover, according to a recent review report, 33.4% of SAC were infected by STHs [6].

In 2020, the World Health Organization (WHO) published revised STH control targets for 2030, aiming to reduce the prevalence of heavy and moderate STH infections to less than 2%. Its adoption depends on the prevalence of STHs in the area [7]. The success of this STH control program could be tracked by applying highly sensitive and reliable diagnostic laboratory methods. Accuracy of diagnostic methods is critical for the effectiveness of disease elimination and community-level control in endemic areas. In 2003, the WHO special program for research and training in tropical diseases developed a set of criteria for the ideal test that may be utilized at all levels of the developing world's health care system to guide treatment and clinical management decisions for infectious tropical diseases. These requirements have become widely accepted as an ideal test should be ASSURED (affordable, sensitive, specific, user-friendly, rapid, equipment-free, and deliverable to end-users) [8, 9].

Several diagnostic techniques have been used for the diagnosis of STHs, including direct wet mount, Kato–Katz (KK), spontaneous tube sedimentation technique (STST), McMaster (MM), formol-ether concentration, agar plate culture, and immunodiagnostic and molecular techniques. However, their sensitivity, cost, simplicity, and applicability vary [9, 10]. The KK and MM techniques are mainly recommended for the detection and quantification of STH infections though they have some limitations [9, 11].

Despite its limited sensitivity, the direct wet mount is still employed as a routine clinical diagnostic method for STH infections in Ethiopia. Due to this, STH infections are improperly diagnosed and cases are not managed effectively. Hence, updating and adopting better sensitive, specific, and cost-effective diagnostic methods as routine diagnostic tests for STHs in endemic areas is urgently needed. Therefore, this study aimed to assess the performance of KK, MM, and STST against the composite reference in detecting STHs in stool sample in the Amhara National Regional State.

## 2. Materials and Methods

### 2.1. Study Design, Area, and Period

An institution-based cross-sectional study was conducted among selected primary schools in Amhara Region from May to July 2022 to evaluate the performance of KK, MM, and STST for the detection of STHs. All schoolchildren in selected primary schools, whose ages ranged from 6 to 14 years, who lived for the last six months in the study area prior to data collection, whose parents/guardians gave written consent for their children to participate, and the schoolchild who gave assent and a stool sample were included. Schoolchildren who received any antihelminthic drug within the last three months prior to data collection were excluded from the study.

### 2.2. Sample Size Determination and Sampling Technique

A total of 421 schoolchildren were included in the study. Data were collected in three districts, namely, North Mecha, Bahir Dar City Administration, and Bahir Dar zuria districts. One primary school from each district was randomly selected by the lottery method. Then, study participants were selected by a systematic random sampling technique in each school using class rosters as a sampling frame. The sample size was proportionally allocated in each district and in each school by considering the total number of students.

### 2.3. Data Collection Methods

#### 2.3.1. Questionnaire Data

A structured questionnaire administered through face-to-face interviews was used to collect sociodemographical data. Trained laboratory professionals were engaged in questionnaire-based data collection, stool sample collection, STH detection, and ova quantification.

#### 2.3.2. Stool Collection and Processing

A clean and leak-proof stool container labelled with the participant's unique identification number was given to each study participant and asked to provide approximately 8 grams of a fresh stool sample. Participants were informed to avoid any possible contamination while they were collecting the sample. To avoid any possible sample delay, stool samples collected at Merawi Junner primary school were transported to the Merawi health center laboratory; whereas, stool samples collected at Weramit and Yinesa primary schools were transported to Bahir Dar University, College of Medicine and Health Sciences, and Medical Laboratory Science laboratory within 1 hour of collection. The stool samples were processed and examined by the KK, MM, and STST following standard operating procedures.

#### 2.3.3. Spontaneous Tube Sedimentation Technique (STST)

3 grams of a fresh stool samples was weighed and homogenized in 10 ml of normal saline solution (0.85% w/v). The mixture was filtered through wire mesh into a 50 ml falcon tube, which was then filled with more saline solution up to a 50 ml gauge, plugged, and shaken vigorously. The tube was left to stand for 45 minutes, and then a sample was taken from the sediment, put on a microscope slide, and examined with 10x followed by 40x objectives of a microscope to check for the presence of ova of STH [10].

#### 2.3.4. Kato–Katz

About 2-3 grams of a fresh stool sample was pressed through a mesh screen to remove large particles. About 41.7 milligram of stool was sieved and transferred to the template which was put on a slide until the template hole was filled. Then, the template was removed, and the stool sample was covered and pressed with cellophane, which was previously immersed overnight in glycerol-malachite green solution. The KK smears were examined within 30–60 minutes for hookworm species and after one hour for other STH species [12].

#### 2.3.5. McMaster

Approximately, 2 grams of fresh stool was suspended in a 30 ml of saturated sodium chloride salt solution at room temperature (density 1.2). The faecal suspension was filtered through a wire mesh to remove large debris and mixed 10 times by using Pasteur pipette. Then, 0.15 milliliter aliquot was added to each side of a MM slide chamber. After two minutes of settling, both chambers were examined under light microscope with 10x objectives. The fecal egg count per gram of stool was calculated by multiplying the number of eggs counted by 50 [11].

### 2.4. Performance Evaluation of Diagnostic Methods

The performance of each test was evaluated by its sensitivity (SN), specificity (SP), positive predictive value (PPV), and negative predictive value (NPV) calculated against the composite reference as a “gold” standard. The diagnostic agreements of methods were evaluated by Kappa value, the number of observed agreement, the number of agreements expected by chance, and standard error of the Kappa. The Kappa value was interpreted as follows: 0 indicates no agreement; 0.01–0.20 indicates none to slight agreement; 0.21–0.40 indicates fair agreement; 0.41–0.60 indicates moderate agreement; 0.61–0.80 indicates substantial agreement; and 0.81–1.00 indicates almost perfect agreement [13].

### 2.5. Data Quality Control

Reliability of the study findings was ensured by applying quality control measures to the whole process of the laboratory work (preanalytical, analytical, and postanalytical quality control steps). The investigator has conducted close follow-up and has been engaged during the process of data collection and laboratory examination. Training was given for data collectors and laboratory examiners on the objective of study and laboratory diagnosis of STHs by KK, MM, and STST. The label of the stool cup and the amount of stool sample were checked during sample collection. To eliminate observer bias, smeared slides were examined independently by two laboratory technologists and 10% of the total slides were randomly selected and read by the principal investigator. Results of their observation were recorded separately for later comparison. All discordant results were rechecked by the principal investigator.

### 2.6. Data Analysis

Data were entered into Epi Info version 3.1 and analyzed by using the SPSS version 25 software packages. The results were summarized using tables. Descriptive statistics were used to compute the detection rate of each diagnostic test and the combined result. The SN, SP, NPV, and PPV for each diagnostic method in STH detection were calculated against the combined results as a “gold” standard method. The Kappa values were estimated at 95% CI to determine the strength of agreement of the diagnostic methods.

## 3. Results

### 3.1. Sociodemographic Characteristic and Prevalence of Soil-Transmitted Helminths

A total of 403 schoolchildren participated in this study and provided stool samples, with a 95.7% response rate. Out of these, 223 (55.3%) were males. The mean age of participants was 10.8 ± 2.1 SD years, and most of the study participants (61.3%) were in the age group of 11–14 years. Most of the study participants 308 (76.4%) were rural dwellers. The overall prevalence of STH infection among schoolchildren in the study area was 32.8%. A prevalence of 30.5%, 30.3%, and 38.5% were obtained in Bahir Dar city administration, North Mecha and Bahir Dar zuria districts, respectively (Table 1).

### 3.2. Detection Rates of Soil-Transmitted Helminths by Different Diagnostic Methods

In a single diagnostic method, high detection rate of STH was found by STST, 30.5% (95% CI: 26.1–35.3), followed by MM, 30% (95% CI: 25.6–34.8), and KK 28.5% (95% CI: 24.2–33.2). The most common STH detected was hookworm, 28.5% (115/403), followed by *A. lumbricoides* 3.7% (15/403) (Table 2). The detection rate of *A. lumbricoides* using the STST method was also 1.88 and 1.2 times more sensitive than the MM and KK methods, respectively, but less sensitive than MM in the detection of hookworm.

### 3.3. Performance of Diagnostic Methods to STHs

By using a combination of all three diagnostic methods as a composite reference, higher SN of 93.2% (95% CI: 87.5–96.8) and NPV of 97.1% (95% CI: 94.7–98.4) in STH detection was obtained by STST, followed by MM with an SN of 91.7% (95% CI: 85.6–95.6) and an NPV 96.5% (95% CI: 94.1–98.0). The sensitivity of MM 97.4% (95% CI: 92.6–99.5) in the detection of hookworm was higher than that of STST 92.2% (95% CI: 85.7–96.4) and KK 87.8% (95% CI: 80.4–93.2) (Table 3). Almost perfect agreements of STST (0.948), MM (0.937) and KK (0.901) were obtained with the composite reference. Similarly, nearly perfect agreement was obtained when the two test methods were compared with each other (Table 4).

## 4. Discussion

Accurate diagnostic methods are required for helminthic infection diagnosis, as well as for monitoring treatment efficacy and success of intervention programs. In endemic areas, the lack of a “gold” standard diagnostic method leads to underdiagnosis and/or underreporting of STHs [14]. The current study assessed the performance of three different parasitological diagnostic methods in stool sample (KK, MM, and STST) for STH, using the composite result as a “gold” standard method.

The prevalence of STHs in the present study was 32.8% (95% CI: 28.2–37.8), which is consistent with previous findings of 31% [15] and 38.0% [16] in the Amhara region. The present prevalence was lower than the previous results of 50.0% in Kola Diba, northwest Ethiopia [17] but higher than the 10.8% prevalence obtained in Debre Markos [18]. The difference might be due to variation in environmental sanitation, endemicity of parasites, sample size, and diagnostic methods.

The detection rate of STHs by KK in the present study was 28.5% (95% CI: 24.2–33.2), which was lower than previous report of 36.0% obtained from Tachgayint district, Amhara region [19] and 44.05% in Kenya [20]. However, the current finding is higher than that of previous reports of 16.3% prevalence in Bibugn, Amhara [21] and 16.3% in northeast India [22]. The variation might be due to differences in sample size, geographical area covered and level of environmental sanitation.

The STST is simple to perform, requiring less equipment and detecting a wide range of species [10]. The STST method had a detection rate of 30.5% (95% CI: 26.1–35.3) for STHs, which is consistent with a previous cross-sectional study conducted in the Amhara region [23].

The MM is an easy and fast method and has been extensively used in veterinary parasitology and even in human studies for the estimation of anthelmintic cures [24]. The present finding demonstrated that MM had 27.8% detection rate to hookworm, which is lower than that of an earlier report (63.24%) in the Amhara region [25], but higher than (18.4%) that of previously reported in Brazil in the detection of hookworm [26]. This variation might be due to differences in endemicity of hookworm species.

Diagnostic methods have different sensitivity in the diagnosis of STHs [27]. In the present study, the sensitivity of STST to STH infections was 93.2% (95% CI: 87.5–96.8), which is higher than previously reported (79.2%) sensitivity in the Amhara region [25]. On the other hand, the sensitivity of MM (91.7%) and KK (87.1%) to STHs in the present study are comparable with the previous sensitivity report in Tanzania [28], but there is also a lower sensitivity report to KK (62.4%) and MM (80.0%) than that of the present findings in the detection of STHs in Wondo Genet, Southern Ethiopia [29]. Although STST is simple to apply and has better sensitivity to STH detection than KK and MM, it is not used as a routine diagnostic method in the place of direct saline microscopy and formol-ether concentration methods, especially in STH endemic countries.

The agreement of KK (0.901), MM (0.937), and STST (0.948) techniques with the combined results was almost perfect in detecting STHs. The agreement of the STST technique with the combined results in the present study is supported by previous study conducted in the Amhara region [23]. However, the agreement between KK and MM in the present study was higher than that in study findings from north Argentina [30]. Similarly, the test agreement of MM with the combined results in the present study was higher than that of previous findings from the Amhara region [26] in the detection of hookworm species. The variation could be due to the difference in the composite references used as a “gold” standard diagnostic method.

## 5. Conclusions

The detection rates of the KK, MM, and STST methods are comparable. The sensitivity of KK, MM, and STST is high with perfect agreement. Therefore, KK, MM, and STST can be employed as diagnostic methods for STHs. The STST also offers the benefit of identifying protozoan infections in endemic locations.

## Figures and Tables

**Table 1:** Sociodemographic characteristics of schoolchildren attending selected primary schools in Amhara region, northwest Ethiopia, 2022 (*N* = 403).

Variables		Frequency (*n*, %)	STH Pos (*n*, %)
Sex	Male	223 (55.3)	82 (36.8)
	Female	180 (44.7)	50 (27.8)

Age group	6–10	156 (38.7)	30 (18.6)
	11–14	247 (61.3)	102 (41.3)

Residence	Rural	308 (76.4)	105 (34.1)
	Urban	95 (23.6)	27 (28.4)

Study area	North Mecha	155 (38.5)	47 (30.3)
	Bahir Dar city	131 (32.5)	40 (30.5)
	Bahir Dar Zuria	117 (29.0)	45 (38.5)
	Total	403 (100.0)	132 (32.8)

^*∗*^STH = soil-transmitted helminths, Pos = positive.

**Table 2:** Detection rates of KK, MM, STST, and composite reference method to STHs among schoolchildren in the Amhara region, northwest Ethiopia, 2022 (*N* = 403).

Parasite identified	Diagnostic method			
	Kato–Katz (*n*, %)	McMaster (*n*, %)	STST (*n*, %)	Composite reference (*n*, %)
Hookworm	101 (25.1)	112 (27.8)	106 (26.3)	115 (28.5)
*A. lumbricoides*	13 (3.2)	8 (2)	15 (3.7)	15 (3.7)
*T. trichuria*	1 (0.2)	1 (0.2)	2 (0.5)	2 (0.5)
Total STHs	115 (28.5)	121 (30)	123 (30.5)	132 (32.8)
*S. mansoni*	9 (2.2)	—	11 (2.7)	14 (3.5)
*E. histolytica/dispar*	NA	NA	16 (4)	16 (4)
*E. vermicularis*	1 (0.2)	—	3 (0.7)	3 (0.7)
*H. nana*	1 (0.2)	2 (0.5)	3 (0.7)	3 (0.7)
Total parasite	126 (31.3)	123 (30.5)	156 (38.7)	168 (41.7)

**Table 3:** Diagnostic performance of KK, MM, and STST in STH detection against the composite reference as a “gold” standard in Amhara region, northwest Ethiopia, 2022 (*N* = 403).

Diagnostic methods		STHs		Composite reference as a “gold” standard				*K* value	*P value*
		Pos	Neg	SN (95% CI)	SP (95% CI)	PPV	NPV (95% CI)		
		(*N*)	(*N*)						
KK	Pos	115	0	87.1 (80.2–92.3)	100 (98.6–100)	100	95.1 (92.6–96.8)	0.901	≤0.001
	Neg	17	271						
MM	Pos	121	0	91.7 (85.6–95.6)	100 (98.6–100)	100	96.5 (94.1–98.0)	0.937	≤0.001
	Neg	11	271						
STST	Pos	123	0	93.2 (87.5–96.8)	100 (98.6–100)	100	97.1 (94.7–98.4)	0.948	≤0.001
	Neg	9	271						

*Hookworm*									
KK	Pos	101	0	87.8 (80.4–93.2)	100 (98.7–100)	100	95.4 (92.7–97.1)	0.912	≤0.001
	Neg	14	288						
MM	Pos	112	0	97.4 (92.6 99.5)	100 (98.7–100)	100	98.9 (96.9–99.6)	0.982	≤0.001
	Neg	3	288						
STST	PosNeg	106	0	92.2 (85.7–96.4)	100 (98.7–100)	100	97.0 (94.5–98.4)	0.944	≤0.001
		9	288						

^*∗*^KK = Kato-katz, MM = McMaster, Neg = negative, NPV = negative predictive value, Pos = positive, PPV = positive predictive value, *K* = Kappa, Se = sensitivity, Sp = specificity, STST = spontaneous tube sedimentation.

**Table 4:** Diagnostic agreement between any of the two methods (KK, MM, and STST) used for the detection of STHs from stool specimens attending selected primary schools in Amhara region, northwest Ethiopia from May to July, 2022 (*N* = 403).

Methods		STST		*K*-value	NOA (*N*, %)	NAEC (*N*, %)	SEK	*χ*2	*p* value
		Pos	Neg						
KK	Pos	111	4	0.905	387 (96.0)	235.2 (58.3)	0.023	330.563	≤0.001
	Neg	12	276						
MM	Pos	114	7	0.906	387 (96.0)	232.8 (57.8)	0.023	330.813	≤0.001
	Neg	9	273						

*MM*									
KK	Pos	108	7	0.880	383 (95.0)	236 (58.6)	0.026	312.628	≤0.001
	Neg	13	275						

^*∗*^NOA = number of observed agreements; NAEC = number of agreements expected by chance; SEK = standard error; *K* = Kappa; Pos = positive; Neg = negative.

## Data Availability

The data should also available to Mr. Getaneh Alemu semantic scholar address “https://www.semanticscholar.org/author/Getaneh-Alemu/4671679”.

## Abbreviations

KK: Kato–Katz
MM: McMaster
NPV: Negative predictive value
PPV: Positive predictive value
PSAC: Preschool-aged children
SAC: School-aged children
SN: Sensitivity
SP: Specificity
STH: Soil-transmitted helminth
STST: Spontaneous tube sedimentation technique
WHO: World Health Organization.
